# Establishment of a pipeline to analyse non-synonymous SNPs in *Bos taurus*

**DOI:** 10.1186/1471-2164-7-298

**Published:** 2006-11-26

**Authors:** Michael A Lee, Orla M Keane, Belinda C Glass, Tim R Manley, Neil G Cullen, Ken G Dodds, Alan F McCulloch, Chris A Morris, Mark Schreiber, Jonathan Warren, Amonida Zadissa, Theresa Wilson, John C McEwan

**Affiliations:** 1 AgResearch, Invermay Agricultural Centre, Mosgiel, Private Bag 50034, New Zealand; 2 AgResearch, Molecular Biology Unit, Department of Biochemistry, University of Otago, Dunedin, New Zealand; 3 AgResearch, Ruakura Research Centre, PB3123, Hamilton, New Zealand; 4 Heron Evidence Development Ltd, The Spirella Building, Letchworth Garden City, SG6 4ET, UK; 5 Novartis Institute for Tropical Diseases, 10 Biopolis Rd, #05-01 Chromos, 138670, Singapore

## Abstract

**Background:**

Single nucleotide polymorphisms (SNPs) are an abundant form of genetic variation in the genome of every species and are useful for gene mapping and association studies. Of particular interest are non-synonymous SNPs, which may alter protein function and phenotype. We therefore examined bovine expressed sequences for non-synonymous SNPs and validated and tested selected SNPs for their association with measured traits.

**Results:**

Over 500,000 public bovine expressed sequence tagged (EST) sequences were used to search for coding SNPs (cSNPs). A total of 15,353 SNPs were detected in the transcribed sequences studied, of which 6,325 were predicted to be coding SNPs with the remaining 9,028 SNPs presumed to be in untranslated regions. Of the cSNPs detected, 2,868 were predicted to result in a change in the amino acid encoded. In order to determine the actual number of non-synonymous polymorphic SNPs we designed assays for 920 of the putative SNPs. These SNPs were then genotyped through a panel of cattle DNA pools using chip-based MALDI-TOF mass spectrometry. Of the SNPs tested, 29% were found to be polymorphic with a minor allele frequency >10%. A subset of the SNPs was genotyped through animal resources in order to look for association with age of puberty, facial eczema resistance or meat yield. Three SNPs were nominally associated with resistance to the disease facial eczema (P < 0.01).

**Conclusion:**

We have identified 15,353 putative SNPs in or close to bovine genes and 2,868 of these SNPs were predicted to be non-synonymous. Approximately 29% of the non-synonymous SNPs were polymorphic and common with a minor allele frequency >10%. Of the SNPs detected in this study, 99% have not been previously reported. These novel SNPs will be useful for association studies or gene mapping.

## Background

Single nucleotide polymorphisms (SNPs) are an abundant form of genetic variation in every species. SNPs are more stable than other genetic markers, such as simple sequence repeats although they are not as informative due to their predominantly biallelic nature. They are distributed throughout the genome and in *Homo sapiens *SNPs have been estimated to occur at a frequency of approximately one in every 1,000 bp [1] making them the most common form of genetic variation. SNPs are extremely useful for association studies, gene mapping and phylogenetic studies. They provide genomic landmarks of past events shaping individuals, breeds and species. In species of agricultural importance, SNPs can be used to identify regions influencing important economic traits. Single nucleotide polymorphisms have also been shown to be responsible for a number of economically desirable phenotypes in sheep, such as the muscle hypertrophy phenotype callipyge and the fertility phenotype Inverdale [2,3]. A public SNP database (dbSNP) has been established by the National Center for Biotechnology Information to store sequence variation information from a variety of species [4]. There are currently 19,395 *Bos taurus *SNPs deposited in this database.

SNPs can be identified in coding regions (cSNPs) or non-coding regions. A subset of SNPs in coding regions give rise to variation in the amino acid sequence of the encoded protein and are known as non-synonymous SNPs (nsSNPs). Such SNPs have been reported to occur less frequently than synonymous SNPs, presumably due to evolutionary constraints as selection eliminates deleterious substitutions from the population [5]. Non-synonymous SNPs are therefore of interest as they are more likely to affect the function of the encoded protein and so may influence a phenotype of interest. Indeed, it has been estimated that 20–30% of nsSNPs affect protein function [6,7].

SNPs are very amenable to high-throughput analysis but *in vitro *SNP discovery can be a lengthy and expensive process. Identifying SNPs *in silico *and then validating the polymorphisms *in vitro *provides a cost effective method of SNP discovery for mapping or association studies.

We developed a pipeline for detecting cSNPs and validating these SNPs cost-effectively. Available cattle expressed sequence tagged (EST) sequences were mined to identify cSNPs yielding 15,353 putative transcribed SNPs, of which 6,325 were predicted to be in coding regions. 2,868 of the cSNPs were predicted to be non-synonymous. A subset of the non-synonymous SNPs was genotyped through pools containing DNA from a number of cattle of different breeds and 29% of these SNPs were confirmed to be polymorphic in the animals tested (minor allele frequency >10%). Finally association was calculated between a subset of the validated non-synonymous SNPs and a number of economically important phenotypes.

## Results

In order to detect non-synonymous SNPs 528,218 public bovine expressed sequences were assembled into contigs and polymorphisms in these contigs detected using conservative criteria. In total the region examined encompassed over 11,000 kbp of bovine expressed sequence and resulted in the detection of 15,353 putative SNPs in 7,145 contigs. The sequence of the contigs is given in Additional file 1 and the SNP allele frequencies are listed in Additional file 2. 6,325 of the SNPs were predicted to be coding SNPs and 2,868 of the cSNPs were predicted to result in a change in the amino acid encoded at that position. The severity of the change of amino acid was calculated using an index that combined the scores from a Blosum90 and PAM250 substitution matrix. The putative synonymous and non-synonymous cSNPs are given in Additional file 3. If all base substitutions were equally likely, 76% of the cSNPs would be expected to result in an amino acid change. We found 45.3% of the cSNPs were predicted to be non-synonymous indicating that there is strong selection pressure against amino acid changes in the sequences studied. The ratio of non-synonymous SNPs to synonymous SNPs reported here is in good agreement with the findings of groups who have calculated this ratio from human sequences [5,8].

**Figure 1:**
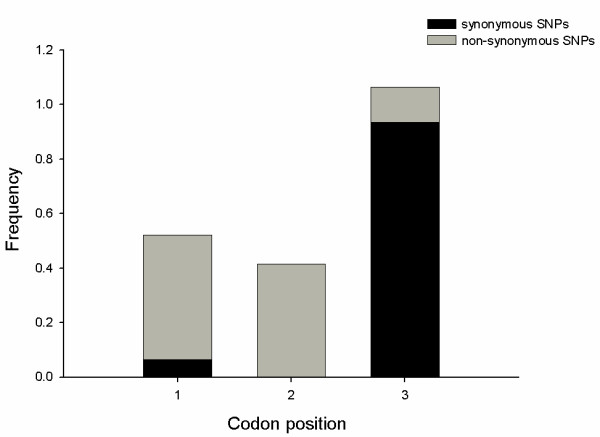
Frequency of substitutions at each of the codon positions for synonymous and non-synonymous SNPs.

**Figure 2:**
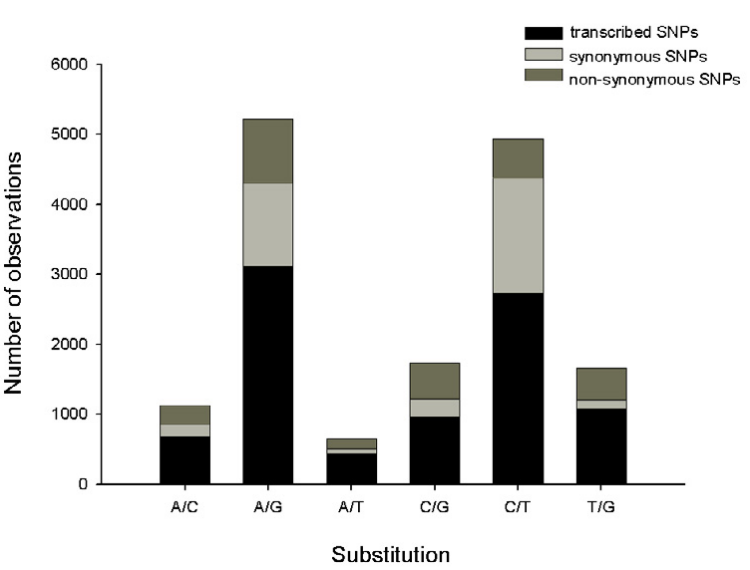
Distribution of transcribed, synonymous and non-synonymous substitutions according to their nucleotide replacement.

The frequency of base substitutions at each of the codon positions for non-synonymous and synonymous SNPs was calculated and is shown in Figure 1. As expected the majority of the substitutions (93.5%) occurred in the third position for synonymous SNPs and at the first and second positions (87.1%) for non-synonymous SNPs. Candidate SNPs were also categorized according to nucleotide substitution as either transitions (A/G or C/T) or transversions (A/C, A/T, C/G, or G/T). Of the 15,353 putative SNPs detected, 15,335 SNPs could be categorized in this manner with the remaining 18 showing 3 alleles at the SNP position. 66.2% of the SNPs detected were the result of transitions, even though transitions represent only one third of the potential types of mutation. A high frequency of transitions has been observed in other SNP discovery programs and the transition frequency observed here is in good agreement with human data where approximately 70% of SNPs were found to be the result of transitions [9-11]. We found the frequency of transitions was significantly higher among synonymous SNPs (81.9%) than non-synonymous SNPs (51.6%; χ^2 ^= 665.8; P = 8.0 × 10^-147^). The frequency of the various substitutions is illustrated in Figure 2. Within the non-synonymous SNPs, the frequency of transitions between conservative substitutions (index score ≥ 2) and radical substitutions (index score ≤ -2) was also examined. In this case no significant difference was found between the two groups (43.9% and 43.8% respectively; χ^2 ^= 0.33; P = 0.97). A high frequency of transitions has been observed in other SNP discovery programs [11] and reflects the high frequency of C to T mutation after methylation.

**Figure 3:**
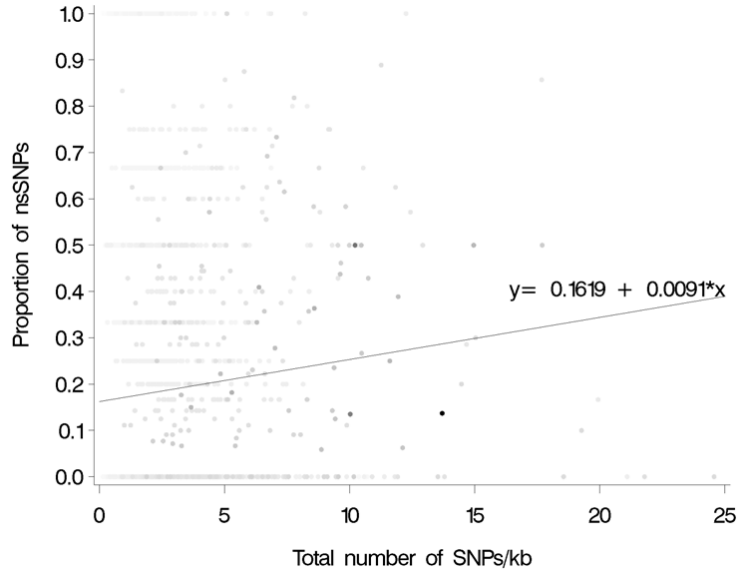
**Scatter plot of proportion of non-synonymous SNPs against SNP density**. The proportion of non-synonymous SNPs is plotted against the total number of SNPs per kb. Contigs were weighted according to the total number of SNPs they contained and this is illustrated by shading (white = 0 to black = 73). A linear relationship exists between the variables given by the equation y = 0.1619 + 0.0091 × (r^2 ^= 0.0071; P < 0.0001).

For each contig, the proportion of nsSNPs was plotted against SNP density and a polynomial regression fitted, weighting by the total number of SNPs the contig contained (Figure 3). Only the linear term was found to explain any effect of SNP density, and this accounted for less than 1% of the variation in the proportion of nsSNPs. Therefore, although the proportion of SNPs that are non-synonymous increases with SNP density, SNP density is a poor predictor of this proportion. The nsSNPs detected in this study appear to come from contigs with a range of SNP densities and are not substantially more frequent in contigs with high sequence variation.

**Figure 4:**
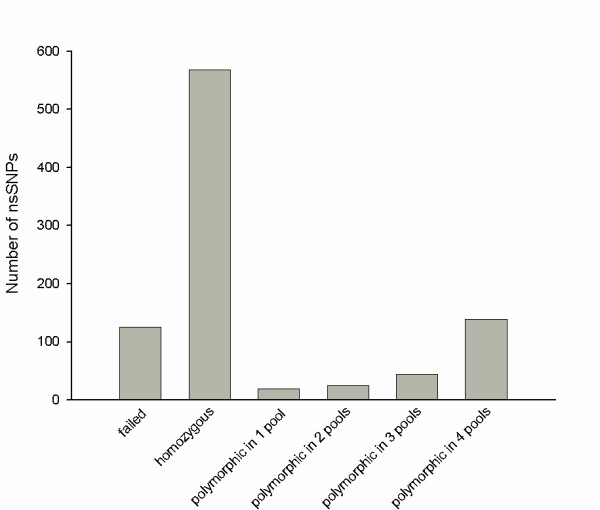
**SNP validation results**. The number of non-synonymous SNPs that failed, were homozygous or were heterozygous for the 920 SNPs genotyped through pooled DNA.

In order to determine the percentage of *in silico *derived non-synonymous SNPs that were polymorphic, 920 nsSNPs were genotyped through pools of cattle DNA using chip based MALDI-TOF mass spectrometry. The pools used consisted of either a single pool containing DNA from 57 animals of a variety of breeds (see methods) or four pools each containing DNA from eight animals from a total of seven breeds plus a back-cross containing two breeds (see Table 1). The pooling strategy provided power to reliably identify SNPs with a minor allele frequency (MAF) greater than 10% and may detect SNPs with a MAF greater than 3%. In total, 795 of the 920 putative nsSNPs tested could be reliably scored giving a strike rate of 86%, while 125 of the assays failed. In total 227 of the putative nsSNPs were confirmed as polymorphic in the animals tested, while 568 of the putative nsSNPs were not confirmed in the animals tested indicating that they are not polymorphic, are rare (MAF <10%), or polymorphism is specific to breeds not tested in this study. The number of nsSNPs that were polymorphic in the pools tested is illustrated in Figure 4. In total 19 SNPs were polymorphic in 1 pool, 25 were polymorphic in 2 pools. 44 were polymorphic in 3 pools and 139 were polymorphic in all 4 pools tested. These 139 SNPs are found in 131 contigs, of which 128 could be annotated with a human RefSeq, giving 112 unique genes. These SNPs represent a subset of highly polymorphic non-synonymous SNPs and include a number of SNPs in immune response genes, such as Major Histocompatibility Complex genes. These genes are known to be extremely polymorphic. The full list of nsSNPs tested and their allele calls is available in Additional file 4.

**Table 1:** Composition of the breed pools.

**Pool**	**Breed**	**Number of animals**	**Total number of animals**
Pool A	Belgian Blue	8	16
	Piedmontese	8	
Pool B	Murray Grey	8	16
	Shorthorn	8	
Pool C	Hereford	8	16
	Simmental	8	
Pool D	Friesian	8	16
	Jersey × Limousin back-cross	8	

As there were a large number of nsSNPs that were polymorphic in all pools tested we needed to eliminate the possibility that this was due to spurious amplification of a closely related gene with a different nucleotide at the extension position. We therefore genotyped 55 nsSNPs, which we scored as polymorphic in all four breed pools, across four individual animals. Only one of these SNPs was heterozygous in all four individual animals tested indicating that amplification of closely related genes is not a serious contaminant in our cSNP discovery program. Approximately 0.8% of the candidate nsSNPs detected in this study were previously deposited in dbSNP. Of the 227 polymorphic nsSNPs validated in this study, 3 had been previously deposited in dbSNP. We have deposited all 15,353 putative SNPs detected in this study in dbSNP.

We tested the utility of a subset of these SNPs to detect by association quantitative trait loci for meat yield, age of puberty and Facial eczema resistance. Facial eczema is a metabolic disease that results from a fungal toxin, sporidesmin, which damages the liver and there is heritable variation in facial eczema resistance in cattle [12]. A total of 41 SNPs in 33 genes with relevance to reproductive traits (6 SNPs in 5 genes), disease resistance (26 SNPs in 19 genes) and body composition (9 SNPs in 9 genes) were genotyped through appropriate animal resources and allele frequencies and association with phenotype calculated. The results are shown in Table 2 and Additional file 5. SNPs in the *BCL10*, *GBP1 *and *C8A *genes were nominally associated with resistance to facial eczema in Jerseys (P = 0.004; 0.006; 0.03 respectively) while SNPs in the *ABCC3*, *OAS1 *and *HRG *genes were nominally associated with resistance to facial eczema in Friesians (P = 0.008; 0.04; 0.05 respectively). There are more nominally significant (P < 0.01) results than expected by chance with 3 exceeding that threshold where less than one would be expected, however, none of the individual SNPs reach a genome wide level of significance. Additionally no SNP was significantly associated with facial eczema resistance in both Jerseys and Friesians.

**Table 2:** SNP allele frequencies and associations with measured traits.

**SNP_ID**	**Gene**	**Breed**	**A**	**C**	**G**	**T**	**P value**
***Puberty***							
CS2000955300001_532	*ANAPC5*	Angus		0.16		0.84	N.S.
CS2000053300001_175	*HMOX2*	Angus			0.58	0.42	N.S.
CS2000507200001_1768	*ITIH2*	Angus	0.41	0.59			N.S.
CS2000742700001_590	*TNFAIP6*	Angus		0.86		0.14	N.S.
CS2001189600001_489	*ITHI5*	Angus	0.84	0.16			N.S.
CS2000742700001_881	*TNFAIP6*	Angus			1		N.S.
***FE resistance***							
CS2000348000001_262	*ABCB8*	Friesian		**0.62**	**0.38**		**N.S.**
		Jersey		**0.63**	**0.37**		**N.S.**
CS2001041800001_1020	*SELP*	Friesian	0.40	0.60			N.S.
		Jersey	0.44	0.56			N.S.
CS2001337000001_147	*CAMP*	Friesian		**0.50**		**0.50**	**N.S.**
		Jersey		**0.51**		**0.49**	**N.S.**
CS2000058700002_510	*CD1D*	Friesian	**0.48**		**0.52**		**N.S.**
		Jersey	**0.49**		**0.51**		**N.S.**
CS2001176100001_1124	*LTB4R*	Friesian			0.16	0.84	N.S.
		Jersey			0.24	0.76	N.S.
CS2000288200001_809	*BCL10*	Friesian		0.93		0.07	N.S.
		Jersey		0.93		0.07	0.004
CS2000088400007_1168	*GBP1*	Friesian		**0.50**		**0.50**	**N.S.**
		Jersey		**0.52**		**0.48**	**N.S.**
CS2001051700001_529	*C8A*	Friesian		0.88		0.12	N.S.
		Jersey		0.75		0.25	0.03
CS2000129600001_1471	*HRG*	Friesian		0.93		0.07	0.05
		Jersey		0.96		0.04	N.S.
CS2001364900001_48	*LTB*	Friesian		0.74	0.26		N.S.
		Jersey		0.65	0.35		N.S.
CS2000088400007_1687	*GBP1*	Friesian	**0.49**			**0.51**	**N.S.**
		Jersey	**0.49**			**0.51**	**N.S.**
CS2000088400007_313	*GBP1*	Friesian		**0.35**		**0.65**	**N.S.**
		Jersey		**0.29**		**0.71**	**0.006**
CS2000088400007_996	*GBP1*	Friesian	**0.50**		**0.50**		**N.S.**
		Jersey	**0.50**		**0.50**		**N.S.**
CS2000257600002_413	*ABCC3*	Friesian		0.76	0.24		0.008
		Jersey		0.77	0.23		N.S.
CS2000313400001_398	*OAS1*	Friesian	**0.23**		**0.77**		**N.S.**
		Jersey	**0.22**		**0.78**		**N.S.**
CS2000348000001_980	*ABCB8*	Friesian			1		N.S.
		Jersey			1		N.S.
CS2001315600002_1764	*SERPING1*	Friesian			0.54	0.46	N.S.
		Jersey			0.56	0.44	N.S.
CS2000173400002_1082	*IGSF8*	Friesian			**0.50**	**0.50**	**N.S.**
		Jersey			**0.73**	**0.27**	**N.S.**
CS2000058700002_496	*CD1D*	Friesian			**0.50**	**0.50**	**N.S.**
		Jersey			**0.50**	**0.50**	**N.S.**
CS2000058700002_976	*CD1D*	Friesian	**0.50**		**0.50**		**N.S.**
		Jersey	**0.48**		**0.52**		**N.S.**
CS2001406800001_259	*TAP2*	Friesian		**0.70**		**0.30**	**N.S.**
		Jersey		**0.73**		**0.27**	**N.S.**
CS2000313400001_155	*OAS1*	Friesian		**0.14**	**0.86**		**0.04**
		Jersey		**0.13**	**0.87**		**N.S.**
CS2000176900001_1229	*HP*	Friesian	0.17		0.83		N.S.
		Jersey	0.35		0.65		N.S.
CS2000045400004_497	*CYP4A11*	Friesian		1			N.S.
		Jersey		1			N.S.
CS2000218500001_1206	*CD44*	Friesian	0.60	0.40			N.S.
		Jersey	0.43	0.57			N.S.
CS2000068700001_485	*GIMAP5*	Friesian	**0.38**		**0.62**		**N.S.**
		Jersey	**0.38**		**0.62**		**N.S.**
***Meat yield***							
CS2000104200001_1729	*PLG*	Angus		0.48		0.52	N.S.
		Hereford		0.53		0.47	N.S.
		Simmental		0.68		0.32	N.S.
CS2000443600001_487	*EPHX2*	Angus	0.98		0.02		N.S.
		Hereford	0.63		0.37		N.S.
		Simmental	0.86		0.14		N.S.
CS2001349700001_285	*ALDOB*	Angus	0.72		0.28		N.S.
		Hereford	0.58		0.42		N.S.
		Simmental	0.43		0.57		N.S.
CS2000071200002_488	*ACADS*	Angus	0.13		0.87		N.S.
		Hereford	0.22		0.78		N.S.
		Simmental	0.14		0.86		N.S.
CS2000660700001_630	*PHYH*	Angus	0.24		0.76		N.S.
		Hereford	0.02		0.98		N.S.
		Simmental			1		N.S.
CS2000613600001_354	*WARS2*	Angus	0.36		0.64		N.S.
		Hereford	0.55		0.45		N.S.
		Simmental	0.18		0.82		N.S.
CS2003511700001_128	*LEP*	Angus		0.40		0.6	N.S.
		Hereford		0.31		0.69	N.S.
		Simmental		0.58		0.42	N.S.
CS2000005800001_509	*ACP1*	Angus		**0.51**		**0.49**	**N.S.**
		Hereford		**0.53**		**0.47**	**N.S.**
		Simmental		**0.50**		**0.50**	**N.S.**
CS2001330700002_10011	*TNXB*	Angus	**0.55**		**0.45**		**N.S.**
		Hereford	**0.49**		**0.51**		**N.S.**
		Simmental	**0.61**		**0.39**		**N.S.**

N.S. = not significant (P > 0.05).

Allele frequencies in bold are not in Hardy-Weinberg equilibrium.

It was found that the allele frequencies of some of the genotyped SNPs were not in Hardy-Weinberg equilibrium. These SNPs are highlighted in bold in Table 2. The reason these SNPs do not obey Hardy-Weinberg equilibrium is unknown, however, it may reflect the fact that the effective population sizes are relatively small, the animals were not randomly mated and the animals genotyped were not a random sample but were chosen as they were outliers in the population.

## Discussion

Using public bovine expressed sequence data we have identified 15,353 candidate SNPs in the genome of *Bos taurus*. One putative SNP was detected approximately every 716 bp for the sequences studied. 6,325 of the putative SNPs are predicted to be coding sequence SNPs and 2,868 of the cSNPs are predicted to be non-synonymous. The low frequency of non-synonymous SNPs detected, with respect to the frequency expected if all base substitutions in coding regions were equally likely, implies that the genes studied are under strong selection pressure. Recently, the deleterious mutation rate was estimated by comparing the coding sequences of many genes between *Bos taurus *and *Homo sapiens*. This analysis concluded that 87% of amino-acid changing mutations were deleterious [13]. Therefore, any non-synonymous SNPs detected in this study may affect the function of the encoded protein. The non-synonymous SNPs we detected were in a variety of genes with differing biological functions and on a number of different bovine chromosomes and may influence traits of economic interest. Additional programs such as SIFT or SNPs3D may be used to predict whether the amino acid substitution will affect protein function [14,15]. However, it is likely that direct association or linkage studies will be needed to determine the real effect of allele substitution on phenotype in an animal or population.

Polymorphisms that affect biological function can also occur outside coding sequences [16], particularly in the proximity of genes such as in splice junctions and promoters. As all of the SNPs detected in this study are in the vicinity of genes, any of these SNPs could potentially influence phenotype or be in linkage disequilibrium with a phenotype-altering mutation. Therefore all SNPs detected in this study are useful for association studies although non-synonymous SNPs are of particular interest.

Approximately 66% of the polymorphisms detected in this study were the result of transitions. Transitions represent only one third of the possible types of substitutions yet they are reported to account for approximately 70% of all substitutions. Our data is in good agreement with previous studies that have calculated the frequency of transitions in human genes [9,10]. The fact that our transition frequency is so close to the expected value implies that our SNP detection strategy is accurate. A high false positive SNP discovery rate would be expected to result in a high frequency of transversions.

The percentage of nsSNPs that were polymorphic was determined by genotyping 920 putative SNPs through pooled DNA from cattle of different breeds. 29% of the SNPs were found to be polymorphic in the samples tested. However, the pooling strategy only allowed reliable detection of SNPs with a minor allele frequency >10%. Therefore many more of the SNPs may be polymorphic, but with a lower MAF. The SNPs were also genotyped through a limited number of cattle breeds, therefore, some SNPs may be present or polymorphic in breeds not tested in this study. Also, many of the ESTs used for SNP detection were derived from cDNA from a limited number of animals, therefore some of the SNPs detected may be restricted to particular animals. Additionally, high-throughput EST sequencing is prone to sequencing errors and this may result in some false positive SNPs. A previous study identifying bovine SNPs from EST data reported a SNP validation rate of 38% for non-synonymous SNPs which had been detected more than once in EST data, although they validated only a small number of SNPs [17]. Therefore, our results are consistent with these findings.

Approximately 0.8% of the non-synonymous SNPs predicted from this study were previously identified and deposited in NCBI dbSNP with the remainder representing novel bovine SNPs. Many of these SNPs could potentially be used as genetic markers in known genes. All SNPs detected in this study and their allele frequencies have been deposited in dbSNP.

Forty one validated polymorphic SNPs in 33 genes of interest were genotyped through animal resources which had been measured for age of puberty, retail beef yield or resistance to facial eczema. The number of nominally significant associations and the strength of these associations with facial eczema resistance was higher than expected by chance although no single SNP reached a genome wide level of significance. The most significant SNP was in the gene B cell lymphoma 10 (*BCL10*) and showed association with facial eczema resistance in a Jersey population. *BCL10 *encodes a protein that appears to both activate and be activated by NFκB and overexpression of *BCL10 *causes weak apoptosis mediated via an amino terminal caspase recruitment domain (CARD) [18,19]. The BCL10 protein is a positive regulator of lymphocyte activation and proliferation [20]. It is more likely that this gene is in linkage disequilibrium with a resistance conferring polymorphism in Jerseys, than responsible for facial eczema resistance *per se*, as the SNP in this gene was not associated with facial eczema resistance in Friesians.

## Conclusion

We have developed a pipeline for detecting, validating and genotyping large numbers of single nucleotide polymorphisms using *Bos taurus *as a model organism. However, this strategy could be successfully applied to any organism for which sufficient EST sequence data exists. We have detected 15,353 putative SNPs in the vicinity of genes in this study and 6,325 candidate cSNPs, of which 2,868 were predicted to be non-synonymous. Approximately 0.8% of the non-synonymous SNPs detected in this study were previously deposited in dbSNP while 99% appear to be novel bovine SNPs. These novel SNPs have the potential to add greatly to the previously discovered bovine SNPs although some additional SNP validation work is required. These SNPs can be used for association studies or gene mapping and we have found SNP associations for resistance to facial eczema in Jersey or Friesian populations at a higher rate than expected by chance, although no single SNP reached a genome wide level of significance. Whether these genes are involved with facial eczema resistance in specific breeds, false positives, or are merely in linkage disequilibrium with a resistance enhancing polymorphisms remains to be determined.

## Methods

### Generation of ESTs

Tissue samples were collected from a number of animals both from research farms and commercial farms. These animals were of different ages and stages of development and the tissues that were collected are listed in Additional file 6. Tissue was sent to Genesis Biotechnology (Auckland, New Zealand) or Life Technologies (Carlsbad, California) where cDNA clones were generated and single pass sequenced giving ESTs. All cDNA libraries were non-normalised with the exception of BEMN. All animal handling and procedures were approved by the AgResearch Animal Ethics Committee.

### Contig assembly

Single pass sequences were vector trimmed, quality clipped and masked by Genesis Biotechnology. For the contig assembly all EST sequences were further cleaned by re-checking for possible vector contamination against UniVec using Cross_match. A total of 528,218 public and AgResearch bovine sequences were assembled into contigs after masking using RepeatMasker. The contig assembly used a divide-and-conquer strategy [21] where an initial coarse partitioning of ESTs was performed to generate correct and feasible assembly sets of no more than 50,000. A simple graph based clustering algorithm was used to generate the assembly sets in which each EST is a node and ESTs are joined by an edge if a BLAST alignment exists between them with an e value ≤ 1 × 10^-10^. This value was chosen to ensure that pairs of ESTs with a 40 bp overlap of 80% identity (the relevant CAP3 parameters used for the assembly) would always be connected. Assembly sets correspond to connected components of the graph. Our initial partitioning generated one infeasible assembly set. This set was partitioned into smaller sets, using the MCL graph clustering algorithm [22]. The parameters used for the MCL re-partitioning were -I 2 -adapt. For each assembly set the unmasked sequences were then assembled into contigs using CAP3 [23]. The default parameters of all programs were used unless otherwise stated. The contig generation process is summarised in Table 3. All AgResearch bovine sequences used are publicly available at NCBI and have accession numbers DY037420 – DY223196 and DY588300 – DY588367.

**Table 3:** Statistics of contig generation.

Total number of ESTs in contig assembly	528,218
Total number of AgResearch ESTs in contig assembly	185,845
Total number of public ESTs in contig assembly	342,373
Number of BLAST clusters	36,047
Number of BLAST singletons	53,493
Number of contigs generated by CAP3	43,117
Total number of singletons (BLAST + CAP3)	66,738
Total number of ESTs available for SNP detection (≥4 per contig)	427,629
Total number of contigs available for SNP detection	22,994

### Contig annotation

Contigs were annotated with their top human RefSeq (3/2006) hit using discontiguous Mega BLAST and the following options **-m 8 -D 2 -t 21 -W 11 -q -3 -r 2 -G 5 -E 2 -e 1e-5 -N 2 -F "m D" -U T -v 5 -b 5. **Discontiguous Mega BLAST, which is based on the use of discontiguous templates [24], is designed for alignments of diverged sequences, most notably sequences from different organisms. The options used here are optimised for alignments where the percent sequence identity is typically 91% [25]. The options specify a template 21 bp long of which 11 positions must be identical to the template match. The options align both coding and non-coding sequences and do not allow the initiation of seeds from the template match in low complexity or repetitive regions, although they do allow extension through these regions.

### cSNP detection

A SNP detection program (SNooPy) was developed to detect single nucleotide polymorphisms in the EST sequences using stringent criteria. SnooPy retrieves contigs with four or more constituent ESTs. Positions with polymorphisms that fulfil the following criteria are then identified: minor allele frequency (MAF) must equal at least 15% or two divided by the contig depth (whichever is greater); there must be at least four aligned ESTs in the region containing the polymorphism; SNPs are disqualified if more than 5% variation is found in the surrounding 10 bp; no more than three variants at a single position are allowed and SNPs detected in the first or last 20 bp of the assembled sequences were not allowed. Synonymous and non-synonymous cSNPs were detected by initially manually blasting the contigs in which putative SNPs had been identified against SWISS-PROT using the following BLAST options **-e 1e-5 -F "m S" -v 50 -b 50**. The results of the BLAST, along with the initial contig sequence, were then evaluated by calculating the frame of the contig sequence translation using programs SNP_SEQ and PROTEIN and hence identifying all cSNPs, including those that result in a change in the amino acid sequence. An index score for the severity of the amino acid change was calculated by adding the PAM250 substitution matrix score and the BLOSUM90 substitution matrix score. The SNooPy, SNP_SEQ and PROTEIN programs are available on request from the corresponding author.

### Validation of non-synonymous SNPs

All human exon sequences, along with 100 bp on either side, were extracted from ENSEMBL. These exon sequences were stored in a BLAST database. Contigs containing putative non-synonymous SNPs were then blasted against this database in order to detect intron-exon boundaries flanking the SNP. As intron-exon boundaries have been shown to be highly conserved between mammalian sequences [26] the cattle exon boundaries were inferred from the human boundaries. Only SNP-containing sequences, predicted to be from single exons with >25 bases flanking the SNP were used in assay design. In order to determine the percentage of *in silico *derived putative nsSNPs that were polymorphic 920 candidate nsSNPs were genotyped through pooled cattle DNA. 268 SNPs were genotyped 4 times (pools A-D) through a single pool containing 57 animals. These animals consisted of 4 Angus, 4 Simmental, 4 Hereford prime, 4 Hereford, 4 Limousin, 4 Murray Grey, 4 Belgian blue, 4 polled Herefords, 4 Shorthorn, 4 Red Devon, 4 Charolais, 4 Piedmontese, 5 Jersey and 4 Jersey × Limousin backcross animals. The SNPs genotyped in this manner are highlighted in bold in Additional file 4. The remaining 652 putative nsSNPs were genotyped through four breed pools of cattle samples (Table 1). All SNPs were genotyped using the MassEXTEND assay (Sequenom). Forward and reverse amplification primers and an extension primer were designed in the region of each putative SNP using SpectroDESIGNER™ 1.3.4 (Sequenom). The target region was amplified with these gene specific primers (Additional file 4). Amplification was carried out with the HotMaster PCR kit (Eppendorf) as follows; 94°C for 1 min, 35 cycles of 94°C for 30 sec, 58°C for 30 sec, 65°C for 45 sec, then 65°C for 3 min. After PCR amplification, unincorporated dNTPs were removed by shrimp alkaline phosphatase. A detecting primer immediately adjacent to the polymorphic site was added together with a specific combination of deoxy and di-deoxy nucleotides (Additional file 4) and thermosequenase (GE Healthcare). The extension products were then analyzed by mass spectrometry on a Biflex 2 MALDI-TOF (Sequenom). For the single pool genotyped 4 times, SNPs were considered polymorphic if at least 1 pool showed the minor allele with a peak height ≥ 10% of the major peak. For the breed pools SNPs were considered polymorphic if at least 1 pool showed the minor allele with a peak height ≥ 12.5% of the major peak. This allowed us to detect SNPs with a MAF of 3–10%. These thresholds were chosen as the peak height of the minor allele was indistinguishable from background when it was < 10% of the major allele peak height. 55 polymorphisms were confirmed by genotyping 4 animals individually (3 Jersey × Limousin animals and 1 Friesian) using MassEXTEND as described above.

### DNA extraction and generation of pools

All genomic DNA was extracted from 10 ml of whole blood or from frozen semen using saturated NaCl as described previously [27]. DNA was quantified using a NanoDrop^® ^ND-1000 Spectrophotometer (NanoDrop Technologies). Pools were created by mixing equimolar amounts of DNA samples to a final concentration of 5–10 ng/μl.

### Association studies

In total 41 SNPs, in 33 genes were genotyped across resources that had phenotypes measured for traits of economic importance. The SNPs were multiplexed into 4 assays and primers designed using MassARRAY Assay design V3.0 (Sequenom). The SNPs were genotyped across the appropriate resources using the iPLEX system (Sequenom) according to the manufacturer's instructions. The full list of multiplexed genotyped SNPs and primers used is available in Additional file 7.

### Resource populations

#### Disease resistance

This population consisted of 112 Jersey-sired and 53 Friesian-sired animals that are facial eczema resistant and 104 Jersey-sired and 54 Friesian-sired animals that are facial eczema susceptible. Resistance or susceptibility to facial eczema was calculated by measuring activity of the liver-derived serum enzyme gamma-glutamyl transferase (GGT) as an indicator of liver injury from facial eczema. GGT levels were measured either after orally dosing the animals with the naturally occurring sporidesmin toxin or after natural challenge at pasture and high and low outliers were chosen [12].

#### Percentage meat yield in the carcass

This population consisted of 349 animals from a variety of breeds, which had been previously identified as having high or low retail beef yield percentage. The number of animals from each breed and their method of selection are listed in Table 4.

**Table 4:** Number and breed of animals chosen for retail beef yield percentage association studies.

**Breed**	**Number of Animals**	**Distribution and Selection Method**
Angus	49	23 high + 26 low - Breeding value^1^
Hereford	70	59 high + 11 low - Breeding value^1^
Simmental	30	26 high + 4 low - Breeding value^1^
Angus weight selection line	40	20 high + 20 control - Genetic selection^2^
NZ Jersey × Limousin	3	3 segregating sires which were backcrossed to Jersey or Limousin dams to produce the progeny below
NZ Jersey × Limousin 3:1	21	10 high + 11 low - Carcass dissection^3^
NZ Jersey × Limousin 1:3	42	21 high + 21 low - Carcass dissection^3^
Australian Jersey × Limousin	3	3 segregating sires which were backcrossed to Jersey or Limousin dams to produce the progeny below
Australian Jersey × Limousin 3:1	21	11 high + 10 low - predicted % meat^4^
Australian Jersey × Limousin 1:3	42	21 high + 21 low - predicted % meat^4^
Angus puberty selection line	3	3 F1 segregating sires
Angus puberty selection line (AGE+)	7	progeny from 2 sires (3 high + 4 low for yield)^5^
Angus puberty selection line (AGE-)	18	progeny from 2 sires (9 high + 9 low for yield)^5^

^1 ^Breeding values calculated from Breedplan (Industry database of NZ animals).

^2 ^Genetic selection for live weight based on [30].

^3 ^Adjusted % meat from carcass dissection of one side of each animal.

^4 ^Predicted % meat yield from meat cuts retrieved after slaughter.

^5 ^[28, 31].

#### Reproductive traits

This population consisted of 95 Angus animals, of which 46 were from an AGE+ (increased age at puberty in heifers) selection line and 49 were from an AGE-(reduced age at puberty in heifers) selection line [28].

### Association testing

#### Disease resistance

For facial eczema, association with each SNP was tested by fitting breed of animal and each SNP genotype nested within breed in a model against standardised log_e _GGT. Standardising was performed on a within-herd/group basis to account for herd/group effects of natural and artificial challenges of sporidesmin.

#### Body composition

Animals with outlier phenotypes for retail beef yield were from 6 sources/breeds. For Angus, Hereford and Simmental, Breeding Values calculated by Breedplan were used and for the other three sources, standardised residuals after fitting fixed effects such as slaughter group, age at slaughter and cohort (generally birth year, sex and pre-slaughter grazing mob) were tested for association by fitting each SNP within source/breed.

#### Reproductive traits

There were two sex-related traits for the puberty dataset; average scrotal circumference over 3 to 6 monthly measurements around puberty in males and a standardised age at first oestrus for females. Animals used were a random subset of males and females born in two years of the selection experiment. Association was tested primarily by looking for greater allelic segregation between the two selection lines than would be expected to arise by genetic drift [29]. Additionally for the two traits, each SNP was fitted within each selection line to test for an association. The two traits were combined using the genetic correlation between the two traits to give one measure for all animals and again tested.

## Authors' contributions

MAL designed the assays for nsSNP validation and resource animal genotyping and coordinated the study. OMK analysed the properties of the SNPs and prepared the manuscript. TRM and BCG carried out the SNP analyses and assembled the data for analysis. NGC and CAM were responsible for identifying and procuring the DNA samples used. CAM was responsible for developing the Early- and Late-'Age at Puberty' lines of cattle from which outlier animals were DNA-sampled and NGC performed the tests of Hardy-Weinberg equilibrium and association between SNPs and phenotypes. KGD provided statistical support. AFM assembled the contigs and annotated them. MS developed the SNP detection program SNooPy. AZ developed the programs to identify synonymous and non synonymous SNPs. JW determined the intron exon boundaries, the number of SNPs in dbSNP and provided bioinformatics support. TW coordinated the public release of all bovine ESTs, coordinated the study and participated in project analysis and writing. JCM was involved in the design of the bioinformatic pipeline, contig assembly, SNP and nsSNP identification and the analysis, estimation and summarisation of their properties. All authors read and approved the final manuscript.

## Supplementary Material

Additional File 1 Text file containing the FASTA sequence of all contigs containing SNPs with the SNP noted. Click here for file

Additional File 2 Excel spreadsheet listing all putative SNPs and their properties. Click here for file

Additional File 3 Excel spreadsheets listing all putative synonymous and non-synonymous SNPs detected in the bovine sequences analysed. Click here for file

Additional File 4 Excel spreadsheet listing all putative non-synonymous SNPs genotyped through the pools along with the primers used, assay conditions and allele calls. Click here for file

Additional File 5 Word document of genotype means for SNP that had nominally significant associations with facial eczema resistance. Click here for file

Additional File 6 Word document listing the cDNA libraries generated and their source tissue. Click here for file

Additional File 7 Excel spreadsheet listing all non-synonymous SNPs genotyped through resource animals and assay conditions. Click here for file
