# Supplementary material for: Establishment of a pipeline to analyse non-synonymous SNPs in Bos taurus
Source: BMC Genomics. 2006 Nov 26;7:298. doi: 10.1186/1471-2164-7-298 (PMC1684264; doi:10.1186/1471-2164-7-298)
Supplement: Additional File 5 — Word document of genotype means for SNP that had nominally significant associations with facial eczema resistance. [file 1471-2164-7-298-S5.doc]

**Additional file 5. Genotype least square means and standard errors for SNP that had nominally significant associations (P<0.05) with facial eczema expressed as log_e_(GGT) levels**

| **SNP** | **Gene** | **Breed** |  |  |  |  | **Sign** |
| --- | --- | --- | --- | --- | --- | --- | --- |
| CS2000288200001_809 | BCL10 |  | genotype | CC | CT | TT |  |
|  |  | Friesian | estimate | 0.49 ± 0.16 | 0.84 ± 0.45 | 0.41 ± 1.05 | N.S. |
|  |  | Jersey | estimate | 0.33 ± 0.12 | -0.58 ± 0.29 | - | 0.004 |
|  |  |  |  |  |  |  |  |
| CS2000088400007_313 | GBP1 |  | genotype | CC | CT | TT |  |
|  |  | Friesian | estimate | - | 0.73 ± 0.28 | 0.73 ± 0.43 | N.S. |
|  |  | Jersey | estimate | - | 0.39 ± 0.23 | -0.60 ± 0.27 | 0.006 |
|  |  |  |  |  |  |  |  |
| CS2001051700001_529 | C8A |  | genotype | CC | CT | TT |  |
|  |  | Friesian | estimate | 0.54 ± 0.17 | 0.34 ± 0.33 | 0.06 ± 1.06 | N.S. |
|  |  | Jersey | estimate | 0.29 ± 0.15 | 0.21 ± 0.18 | -0.79 ± 0.45 | 0.03 |
|  |  |  |  |  |  |  |  |
| CS2000257600002_413 | ABBC3 |  | genotype | CC | CG | GG |  |
|  |  | Friesian | estimate | 0.86 ± 0.19 | 0.04 ± 0.24 | 0.18 ± 0.66 | 0.008 |
|  |  | Jersey | estimate | 0.02 ± 0.14 | 0.41 ± 0.19 | 0.59 ± 0.43 | N.S. |
|  |  |  |  |  |  |  |  |
| CS2000313400001_155 | OAS1 |  | genotype | CC | CG | GG |  |
|  |  | Friesian | estimate | - | -0.01 ± 0.31 | 0.75 ± 0.19 | 0.04 |
|  |  | Jersey | estimate | - | 0.06 ± 0.24 | 0.26 ± 0.14 | N.S. |
|  |  |  |  |  |  |  |  |
| CS2000129600001_1471 | HRG |  | genotype | CC | CT | TT |  |
|  |  | Friesian | estimate | 0.65 ± 0.16 | -0.27 ± 0.43 | -0.58 ± 1.50 | 0.05 |
|  |  | Jersey | estimate | 0.09 ± 0.11 | 0.45 ± 0.40 | 1.06 ± 1.50 | N.S. |
