# Supplementary material for: Establishment of a pipeline to analyse non-synonymous SNPs in Bos taurus
Source: BMC Genomics. 2006 Nov 26;7:298. doi: 10.1186/1471-2164-7-298 (PMC1684264; doi:10.1186/1471-2164-7-298)
Supplement: Additional File 6 — Word document listing the cDNA libraries generated and their source tissue. [file 1471-2164-7-298-S6.doc]

**Additional file 6. List of cDNA libraries and their source tissue**

| Library | Organ | Tissue | Breed | Sex |
| --- | --- | --- | --- | --- |
| BABA | Brain | Anterior brain stem | Unknown | Unknown |
| BAGA |  | Adrenal gland | Unknown | Unknown |
| BAMA |  | Muscle | Unknown | Unknown |
| BAPA |  | Anterior pituitary | Unknown | Unknown |
| BCEA | Brain | Cerebellum- post natal | Unknown | Unknown |
| BCEB | Brain | Cerebellum – 1 year | Unknown | Unknown |
| BCMA | Heart | Cardiac muscle | Unknown | Unknown |
| BCNA | Ovary | Contralateral ovary – non-ovulated | Friesian | Female |
| BCOA | Ovary | Contralateral ovary - ovulated | Friesian | Female |
| BCXA | Brain | Cortex | Unknown | Unknown |
| BCXB | Brain | Cortex | Unknown | Unknown |
| BEMA |  | Embryonic | Unknown | Unknown |
| BEMN |  | 25% Embryonic, 25% Placenta, 25% Mammary tissue, 25% Immune tissue | Unknown | Unknown |
| BFMA |  | Foetal muscle | Unknown | Unknown |
| BHFA |  | Fat tissue | Angus x Friesian | Female |
| BHTA |  | Hypothalamus | Unknown | Unknown |
| BINA | Ovary | Ipsilateral ovary - non-ovulated | Friesian | Female |
| BIOA | Ovary | Ipsilateral ovary - ovulated | Friesian | Female |
| BLIA | Liver | Liver | Unknown | Unknown |
| BLIB | Liver | Liver | Unknown | Unknown |
| BMAA | Mammary Gland | Mastitic mammary | Unknown | Female |
| BMCA | Mammary Gland | Mastitic mammary gland (mastitis affected cisterna) | Unknown | Female |
| BMGA | Mammary Gland | Mammary (2 hours post milking) | Jersey | Female |
| BMGS | Mammary Gland | Mammary (2 hours post milking) | Jersey | Female |
| BMLB | Mammary Gland | Mammary (late involution) | Angus | Female |
| BMNA |  | Mesenteric lymph node | Friesian x | Unknown |
| BMPA | Mammary Gland | Pre-partum mammary tissue | Angus | Female |
| BMUA | Mammary Gland | Mastitic mammary | Unknown | Female |
| BMVA | Mammary Gland | Mammary tissue - virgin | Friesian | Female |
| BMVB | Mammary Gland | Mammary tissue - virgin | Friesian | Female |
| BOVA | Ovary | Ovarian | Unknown | Female |
| BOVB | Ovary | Ovarian | Unknown | Female |
| BPBA | Brain | Posterior brain stem | Unknown | Unknown |
| BPLA |  | Placenta | Unknown | Female |
| BPLC |  | Placenta | Unknown | Female |
| BPMA |  | Ficoll purified peripheral blood mononuclear cells | Friesian x | Unknown |
| BPMS | Mammary Gland | Subtracted bovine mammary gland (pregnant MG-lactating MG) | Unknown | Female |
| BPMT | Mammary Gland | Subtracted bovine mammary gland (pregnant MG-lactating MG) | Unknown | Female |
| BPNA |  | Prescapular Lymph node | Friesian x | Unknown |
| BPPA |  | Peyers Patch | Friesian x | Unknown |
| BPSA |  | Paratoid salivary | Unknown | Unknown |
| BSIA | Small Intestine | Small intestine mucosa | Friesian x | Unknown |
| BSMA | Muscle | Skeletal muscle | Unknown | Unknown |
| BSPA | Spleen |  | Unknown | Unknown |
| BSPB | Spleen |  | Unknown | Unknown |
| BSVA |  | Seminal vesicle | Unknown | Male |
| BTMA |  | Thymus | Unknown | Unknown |
| BTNA |  | Tonsil | Friesian x | Unknown |
| BTSA | Testes |  | Unknown | Male |
| BTYA |  | Thyroid | Unknown | Unknown |
| BUTA | Mammary Gland | Teat | Unknown | Female |
